# TNF Signaling Acts Downstream of MiR-322/-503 in Regulating DM1 Myogenesis

**DOI:** 10.3389/fendo.2022.843202

**Published:** 2022-04-07

**Authors:** Meng Li, Feng Xu, Zhongxian Liu, Chunguang Wang, Yun Zhao, Guoping Zhu, Xiaopeng Shen

**Affiliations:** ^1^ Anhui Provincial Key Laboratory of Molecular Enzymology and Mechanism of Major Diseases, College of Life Sciences, Anhui Normal University, Wuhu, China; ^2^ Anhui Provincial Key Laboratory of the Conservation and Exploitation of Biological Resources, College of Life Sciences, Anhui Normal University, Wuhu, China; ^3^ Key Laboratory of Biomedicine in Gene Diseases and Health of Anhui Higher Education Institutes, College of Life Sciences, Anhui Normal University, Wuhu, China; ^4^ Hefei Institutes of Physical Science, Chinese Academy of Sciences, Hefei, China

**Keywords:** DM1, miR-322/-503, TNF signaling, myogenesis, myoblast

## Abstract

Myotonic dystrophy type 1 (DM1) is caused by the expanded CUG repeats and usually displays defective myogenesis. Although we previously reported that ectopic miR-322/-503 expression improved myogenesis in DM1 by targeting the toxic RNA, the underlying pathways regulating myogenesis that were aberrantly altered in DM1 and rescued by miR-322/-503 were still unknown. Here, we constructed DM1 and miR-322/-503 overexpressing DM1 myoblast models, which were subjected to *in vitro* myoblast differentiation along with their corresponding controls. Agreeing with previous findings, DM1 myoblast showed remarkable myogenesis defects, while miR-322/-503 overexpression successfully rescued the defects. By RNA sequencing, we noticed that Tumor necrosis factor (TNF) signaling was the only pathway that was significantly and oppositely altered in these two experimental sets, with it upregulated in DM1 and inhibited by miR-322/-503 overexpression. Consistently, hyperactivity of TNF signaling was detected in two DM1 mouse models. Blocking TNF signaling significantly rescued the myogenesis defects in DM1. On the contrary, TNF-α treatment abolished the rescue effect of miR-322/-503 on DM1 myogenesis. Taking together, these results implied that TNF signaling mediated the myogenesis defects in DM1 and might act downstream of miR-322/-503 in regulating the myogenesis in DM1. Moreover, the inhibition of TNF signaling benefiting myogenesis in DM1 provided us with a novel therapeutic strategy for DM1.

## Introduction

Myotonic dystrophy type 1 (DM1) is an autosomal inherited neuromuscular disease caused by aberrant expanded trinucleotide repeats (CTG) in the 3’ untranslated region (3’UTR) of the DMPK gene (1, 2). The copy number of CTG repeats was more than 50 in DM1 patients and less than 37 in healthy individuals (3, 4). The mRNA transcribed from the expanded CTG repeats conjugated DMPK gene in DM1 is called toxic RNA, which leads to abnormal expression of MBNL1 and CELF1. As MBNL1 and CELF1 are RNA alternative splicing regulators, DM1 is characterized by aberrant alternative splicing events, which directly cause the pathological phenotypes of DM1, for example, defective myogenesis. Although many mechanisms and pathways have been revealed to regulate myogenesis in DM1, there were still controversies.

Myogenesis is a complicated process that is precisely controlled by multiple regulatory factors and signaling pathways (5). Myogenesis from myoblasts is consists of three steps: cell cycle exit, cell alignment, and cell fusion. Upon myogenesis initiation, Pax7^+^ satellite cells are activated and proliferate to form MyoD^+^ myoblasts (5). MyoD^+^ myoblasts are highly proliferative and their division gives rise to a sufficient number of myogenic progenitors for subsequent steps (6). Next, myoblasts exit the cell cycle and start to differentiate into myocytes. At this stage, MyoG drives myocyte generation, with the expression of MyoD dropping gradually (7). Following that, mononuclear myocytes undergo cell alignment and cell fusion to generate multinucleated myotubes, where Mrf4 and Mef2C function as key regulators (8, 9). In addition, Myomixer and Myomaker are essential factors for the cell fusion process (10). As a result of the whole myogenesis process, Myh3^+^/Myh7^+^ myotubes are produced (11).

MicroRNAs are a group of 18~22 nucleotide non-coding RNAs, which can suppress gene expressions by incorporating the RNA-induced silencing complex (RISC) to the 3’UTR of mRNAs. miR-206, miR-148a, miR-322/-503 clusters were known as important regulators in DM1 and the manipulation of their expressions could be potential therapeutic approaches for DM1 defects (12, 13). Specifically, miR-206 and miR-148a targeted the non-CUG repeat region of DMPK 3′UTR, while miR-322/-503 directly targeted both Celf1 and the toxic RNA. Moreover, the ectopic miR-322/-503 expression could significantly improve myogenesis in DM1, whereas leaving the underlying mechanisms or pathways that mediated such function unknown.

Tumor necrosis factor (TNF) signaling is a pleiotropic pathway that regulates both homeostasis and disease pathogenesis. In the presence of TNF-α, IKK-α/β activates downstream NF-κB signaling, and the activated NF-κB enters the nucleus to participate in the transcription of Caspase8, Cxcl5, Lif, and other genes, ultimately leading to cell apoptosis and inflammation (14–16). Several miRNAs, such as miR-218, miR-451, and miR-322, were previously reported as fine-tuning regulators of the TNF signaling (17–19). Recent studies suggested that hyperactive TNF signaling led to skeletal muscle atrophy and impaired myogenesis (20, 21). In addition, with TNF-α treatments, miR-322 and miR-335 levels were closely associated with myogenesis extents (22). Nevertheless, in DM1, the effects of TNF signaling on myogenesis and the function of miR-322/-503 were uninvestigated to our best knowledge.

Here we discovered that TNF signaling was hyperactive in both DM1 cell and mice models and miR-322/-503 rescued myogenesis defects in DM1 at least partially through the TNF signaling. Meanwhile, the inhibition of TNF signaling using a chemical inhibitor significantly rescued myogenesis defects in DM1, which implied a potential therapeutic role of TNF signaling.

## Materials And Methods

### Cell Culture

C2C12 cells used in this study were kindly provided by Stem Cell Bank, Chinese Academy of Sciences. The cells were authenticated by STR profiling and free of mycoplasma contamination. C2C12 cells were cultured in the growth medium (high-glucose Dulbecco’s Modified Eagle Medium (DMEM, Gibco, Cat# C11965500BT), supplemented with 20% Fetal Bovine Serum (Clark bioscience, Cat# FB15015), 50 U/mL penicillin, and 50 μg/mL streptomycin (Biosharp, Cat# BL505A)). For normal culture, C2C12 cells were seeded onto 6-well plates (8×10^4^/well) and cultured in the growth medium. C2C12 cells were subcultured when they reached ~70% confluence.

For *in vitro* myoblast differentiation, C2C12 cells were seeded onto 6-well plates (8×10^4^/well) and cultured in the growth medium until 80-90% confluence (~9×10^5^/well). Next, C2C12 cells were switched to culture in the differentiation medium (DMEM supplemented with 2% Horse Serum (HyClone, Cat# SH30074.03), 50 U/mL penicillin, 50 μg/mL streptomycin, and 1 μM insulin (Beyotime, Cat# P3376-100IU)). The differentiation medium was changed daily. The culture in the differentiation medium lasted for 6 days. If it is necessary, add a TNF signaling inhibitor, INH14 (100 nM except where otherwise stated, MCE, Cat# HY-114454) or TNF-α (50 ng/ml, Peprotech, Cat# 315-01A) to the medium from day 3 to day 6, with DMSO (Diamond, Cat# A100231-0500) or BSA (Sigma, Cat# V900933) as control, respectively. *in vitro*Total RNA samples were collected on day 0, day 1, day 2, day 4, and day 6, and cells on culture dishes were fixed on day 6 for immunofluorescence staining.

### Plasmid and Cell Line Construction

pCDNA3-GFP-(CUG)_5_ and pCDNA3-GFP-(CUG)_200_ plasmids were as previously reported (23). In such plasmids, 5 and 200 CTG repeats were placed at the 3’ UTR regions of the GFP gene, respectively. In this study, the pLL4.0 backbone was generated by replacing a GFP expressing cassette in the pLL3.7 vector with a puromycin encoding fragment. miR-322/-503 overexpression plasmid (pLL4.0-miR-322/-503) was produced by ligating a miR-322/-503 encoding fragment into the pLL4.0 plasmid at an EcoRI site.

Plasmid transfections were performed with the PolyJet transfection reagent (SignaGen, Cat# SL100688) according to the manufacturer’s suggestion. Cell culture mediums were changed 24h after transfection. The transfected cells were subjected to drug selections when stable cell lines were needed. The drugs used in this study include G418 (1mg/ml, Biofroxx, Cat# 1150GR001) and puromycin (1mg/ml, Sangon Biotech, Cat# A610593), which were adopted regarding specific drug resistances carried in the plasmids. All C2C12 derived cell lines were selected stable before *in vitro* myoblast differentiation.

### Total RNA Extraction and Real-Time Quantitative PCR (RT-qPCR)

Total RNA was extracted from the Total RNA Isolation Reagent (Biosharp, Cat# BS259A). Reverse transcription was performed with FastKing kit cDNA (Tiangen, Cat# KR118-02). Quantitative PCR was performed with Powerup SYBR Master Mix (Applied Biosystems, Cat# A2577) using a Roche Light Cycle96 machine. All these procedures were performed according to the manufacturer’s protocols. Gapdh was used as a normalized control. All RT-qPCR primer sequences were provided in the **Table S1**.

### Immunofluorescence

Cells were briefly washed with PBS and then fixed with 4% paraformaldehyde (PFA, Sangon Biotech, Cat# A500684) for 10 min at room temperature. Following fixation, the cells were permeabilized with 0.1% TritonX-100 in PBS for 30 min. Subsequently, the cells were blocked in the blocking solution (10% normal goat serum, 0.1% Triton X-100 in PBS) for 1 h, and incubated with primary antibodies overnight. On the next day, the cells were incubated with fluorescence conjugated-secondary antibodies for 90 min at room temperature and then stained with DAPI for 5 min. Immunostaining images were taken with a Leica DMI8 fluorescence microscope, and analysis was performed by ImageJ software (RRID: SCR_003070) (24). In fluorescent images, the myotube area is the ratio of fluorescence positive area to the whole area. and fusion index is the ratio of nuclei number in the cells with at least two nuclei versus total nuclei number. These antibodies used were as follows: MF20 mAb (1:10, DSHB, Cat# AB_2147781), Ki67 mAb (1:100, Invitrogen, Cat# MA5-14520), Goat anti-Rabbit Alex Fluor 488-conjugated IgG (1:500, Invitrogen, Cat# A11008), Goat anti-Mouse Alex Fluor 555-conjugated IgG (1:500, Invitrogen, Cat# A32727).

### RNA Fluorescence *In Situ* Hybridization (RNA FISH)

RNA FISH was performed as reported previously (13). Cells were fixed with 4% PFA at 4°C for 20 min and then permeabilized with PBS supplemented with 0.5% Triton X-100 and 2 mM ribonucleoside vanadyl complex (RVC, Beyotime, Cat# R0107) for 7 min. Next, the cells were incubated with 30% formamide (Sangon Biotech, Cat# A600212) and 2× SSC (Sangon Biotech, Cat# B548110) for 10 min. Hybridization was conducted by incubating the cells with the hybridization buffer (30% formamide, 2× SSC, 0.02% bovine serum albumin (Sigma, Cat# V900933), 66 ug/ml yeast tRNA (Invitrogen, Cat# AM7119), 10% dextran sulfate (Sangon Biotech, Cat# A600160), 2 mM RVC, and 2 ng/μl (CAG)_7_ probe) for 24 h. The cells were then washed with 30% formamide and 2× SSC at 45°C for 30 min and then 1× SSC at 37°C for 30 min. Finally, the cells were mounted with Antifade Mounting Medium with DAPI (Beyotime, Cat# P0131) and observed with a Zeiss ApoTome.2 fluorescence microscope. The (CAG)_7_ probe was 5′-CAGCAGCAGCAGCAGCAGCAG-3′ with 5′-FAM label and 2′-O-methyl modification at the first two nucleotides.

### Apoptosis Assay

Apoptosis assay was performed with the Annexin V Apoptosis Detection Kit FITC (BBI, Cat# E606336) according to the manufacturer’s suggestion. Cells were briefly washed with PBS and re-suspended with 1× binding buffer at a density of ~5×10^5^/ml. Subsequently, the cells were incubated with Annexin V-FITC for 15 min at room temperature. Next, the cells were incubated with Propidium Iodide (PI) for 5 min. Finally, the cells were subjected to flow cytometry on a BD FACSCantoII Flow Cytometer (BD). Annexin V^+^ cells were apoptotic cells.

### RNA Sequencing (RNA-Seq)

RNA samples were sent to the Anhui Microanaly Genetech Technology Co., LTD for library construction and RNA-seq. Raw data were first filtered with the Trim-Galore software (RRID: SCR_011847) and then aligned to the mouse GRCm38 genome by the Hisat2 software (RRID: SCR_015530) (25). Next, the Stringtie software (RRID: SCR_016323) was used to generate readcount tables (26). Differentially expressed genes (DEGs) were determined with the DESeq2 software (RRID: SCR_015687) (27), of which the cut-off used was | log2Fold Change | > 1 and adjust p-value< 0.05. Gene ontology (GO) and Gene Set Enrichment Analysis (GSEA) were performed with the clusterProfiler software (RRID: SCR_016884) (28). RNA-seq data generated in this study have been deposited at the Gene Expression Omnibus (GEO) database under the accession numbers GSE174119 and GSE189897. The RNA-seq data of quadricep muscles derived from wild-type and two DM1 mice models were obtained from Sequence Read Archive (SRA) database (PRJNA625451).

### Statistical Analysis

Bar graphs were drawn with the R, Microsoft Excel, and GraphPad Prism 9. The quantification of Immunofluorescence staining images was performed with the ImageJ software. Three biological replicates and three technical replicates were performed for all assays except where otherwise stated. Significance was determined by the student’s t-test and one-way analysis of variance (ANOVA), and p < 0.05 was considered to be statistically significant. All data were presented as mean ± SD.

## Results

### Myogenesis Was Defective in the DM1 Myoblast Model

**Figure 1:**
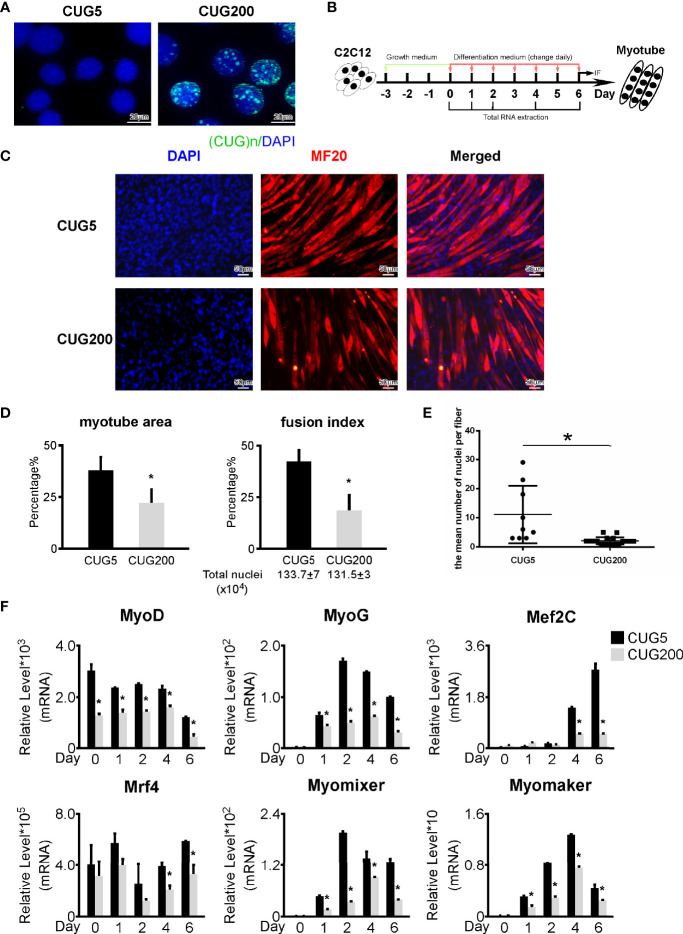
Myogenesis was defective in the DM1 myoblast model. **(A)** Ribonuclear foci were detected in the DM1 myoblast model, but not in the normal group. RNA FISH with the (CAG)7 probe was performed on the normal and DM1 myoblast models. **(B)** Schematic diagram of *in vitro* myoblast differentiation of the normal and DM1 myoblasts. **(C)** Myotube formation was significantly repressed in the DM1 group. Immunofluorescence staining against MF20 was performed on differentiation day 6. **(D)** The myotube area and fusion index were significantly decreased in the DM1 group. **(E)** The mean number of nuclei per fiber was significantly decreased in the DM1 group. **(F)** The expression of muscle regulatory factors (MyoD, MyoG, Mef2C, and Mrf4) and myoblast fusion genes (Myomixer and Myomaker) was significantly downregulated in the DM1 group. CUG5, the normal group; CUG200; the DM1 group; n≥3; *p < 0.05.

We constructed normal and DM1 myoblast models by stably transfecting C2C12 myoblast cells with pCDNA3-GFP-(CUG)_5_ and pCDNA3-GFP-(CUG)_200_ plasmids, respectively. To verify the normal and DM1 myoblast models, we performed RNA FISH with (CAG)_7_ probes as reported previously (13). The DM1 myoblasts showed clear RNA foci, while the normal group did not (**Figure 1A**). We examined the proliferation abilities of these two cell models by immunostaining against Ki67 and found DM1 myoblasts retained a relatively higher proliferation (**Figure S1**). By Annexin V/PI apoptosis assay, we found that DM1 and control myoblasts had similar ratios of apoptotic cells (**Figure S2**). We next performed *in vitro* myoblast differentiation on normal and DM1 myoblasts (**Figure 1B**). By immunostaining against MF20, we observed a severely damaged myotube formation in the DM1 group (**Figure 1C**). Statistical analyses indicated that myotube area and fusion index were both significantly lowered in DM1 (**Figure 1D**). Similarly, the mean number of nuclei per fiber was significantly downregulated in DM1 (**Figure 1E**). By RT-qPCR, we found that myogenesis-related genes (MyoG, MyoD, Mef2C, and Mrf4) and myoblast fusion markers (Myomixer and Myomaker) were all significantly repressed in DM1 (**Figure 1F**). These results suggested that our DM1 myoblast model reproduced the defective myogenesis in DM1 and thus could be applied to further studies on DM1 regulatory mechanisms.

**Figure 2:**
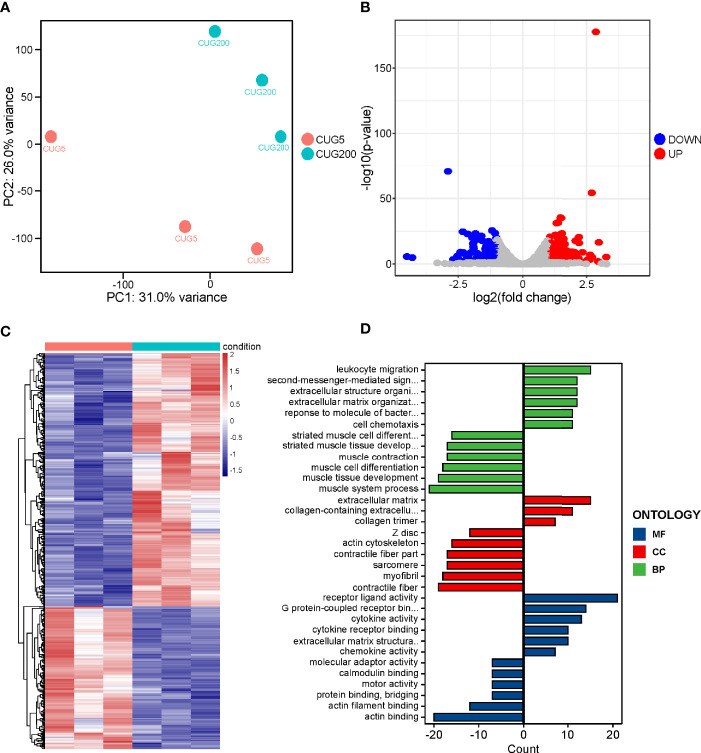
RNA-seq on the differentiating normal and DM1 myoblasts. **(A)** PCA of the RNA-seq data. There were three replicates for each group. **(B)** The volcano plot displayed the distribution of DEGs. Red dots represented the significantly upregulated genes and blue dots represent significantly downregulated genes. |log_2_(Fold Change)| > 1 and adjusted P-value < 0.05 were used as the cut-off value. **(C)** Heatmap showed the relative levels of DEGs between the normal and DM1 groups. **(D)** The GO analysis of DEGs. CUG5, the normal group; CUG200, the DM1 group; MF, molecular function; CC, cellular component; and BP, biological process; n=3.

Previous reports have documented many regulatory factors that might contribute to the defective myogenesis in DM1, such as aberrant alternative splicing and upregulated Celf1, while the underlying pathways that directly mediated this defect remained unclear. To address this, we performed RNA-seq on the normal and DM1 myoblasts that had been subjected to *in vitro* myoblast differentiation on day 4. Principal component analysis (PCA) on this RNA-seq data indicated that the expression patterns of normal and DM1 groups were highly distinct (**Figure 2A**). Through classical DEG analysis, we identified 279 upregulated and 158 downregulated genes using the cut-off of | log_2_Fold Change | > 1 and adjusted p-value< 0.05 (**Figures 2B, C**). Next, we performed GO analysis on these DEGs and found that muscle tissue development-related processes were inhibited, while the processes of cytokine activity, receptor-binding capability, and extracellular matrix (ECM) related processes were significantly strengthened in DM1 (**Figure 2D**). For verification, we performed RT-qPCR on ECM-related factors (Col1a1, Fmod, Postn) and cytokine factors (Cxcl5, Ccl2, IL1β) and found these genes were all significantly upregulated during DM1 myogenesis (**Figure S3**). The alterations of ECM-related gene expression and alternative splicing in DM1 had been studied previously (29), while the cytokines-related processes and pathways in DM1 were previously unrecognized.

### MiR-322/-503 Rescued Myoblast Differentiation Defects

**Figure 3:**
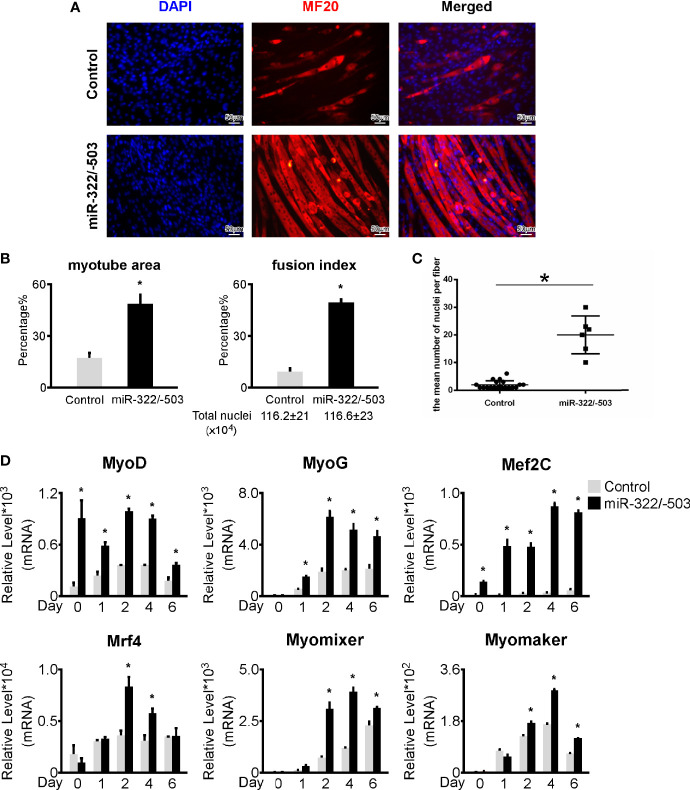
miR-322/-503 rescued myoblast differentiation defects. **(A)** Ectopic miR-322/-503 expression improved myotube formation in DM1 myoblasts. Immunofluorescence staining against MF20 was performed on differentiation day 6. **(B)** The myotube area and fusion index were significantly increased with miR-322/-503 overexpression. **(C)** The mean number of nuclei per fiber was significantly increased with miR-322/-503 overexpression. **(D)** The expression of muscle regulatory factors (MyoD, MyoG, Mef2C, and Mrf4) and myoblast fusion genes (Myomixer and Myomaker) was significantly upregulated with miR-322/-503 overexpression. Control, control empty vector stably transfected DM1 myoblasts; miR-322/-503, miR-322/-503 overexpressing DM1 myoblasts; n≥3; *, p < 0.05.

Our previous work demonstrated that miR-322/-503 could rescue myoblast defects by targeting both the toxic RNA and Celf1 in DM1 (13). In this study, we constructed miR-322/-503 overexpressing the DM1 myoblast cell line. Through *in vitro* myoblast differentiation, we observed a remarkable improvement of myogenesis in DM1 with miR-322/-503 overexpression as indicated by the immunostaining against MF20 and the statistical analyses of myotube area, fusion index, and mean number of nuclei per fiber (**Figures 3A–C**). By RT-qPCR, we found that myogenesis-related genes (MyoG, MyoD, Mef2C, Mrf4) and myoblast fusion markers (Myomixer, Myomaker) were all significantly upregulated (**Figure 3D**). These results confirmed a rescue function of miR-322/-503 on the DM1 myogenesis defect.

**Figure 4:**
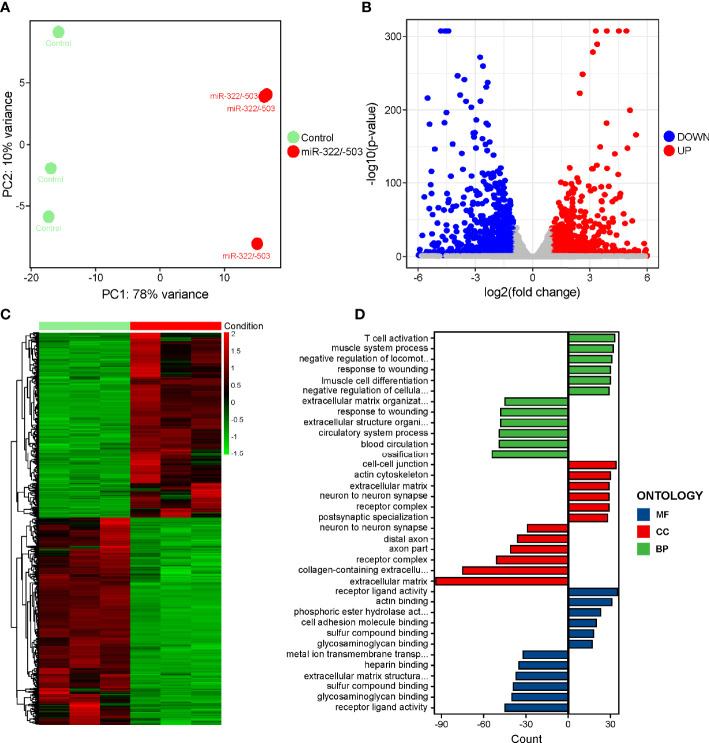
RNA-seq on the differentiating control and miR-322/-503 overexpressing DM1 myoblasts. **(A)** PCA of the RNA-seq data. There were three replicates for each group. **(B)** The volcano plot displayed the distribution of DEGs. Red dots represented the significantly upregulated genes and blue dots represent significantly downregulated genes. |log_2_(Fold Change)| > 1 and adjusted P-value < 0.05 were used as the cut-off value. **(C)** Heatmap showed the relative levels of DEGs between the two groups. **(D)** The GO analysis of DEGs. Control, control empty vector stably transfected DM1 myoblasts; miR-322/-503, miR-322/-503 overexpressing DM1 myoblasts; MF, molecular function; CC, cellular component; and BP, biological process; n=3.

Similarly, to interpret the downstream pathways that mediated the rescue function of miR-322/-503 on DM1 myogenesis defects, we performed RNA-seq on the control and miR-322/-503 overexpressing DM1 myoblasts that had been differentiated on day 4. PCA showed that these two groups were well separated along with the principal component 1 (PC1) division, which accounted for 78% of the variation in gene expression (**Figure 4A**). Through DEG analysis, we identified 941 upregulated and 1053 downregulated genes (**Figures 4B, C**). GO analysis indicated that muscle development processes were strengthened with miR-322/-503 overexpression, while the cytokine receptor activity process was inhibited (**Figure 4D**). Combining with the notion that the cytokines and their receptors related processes were upregulated in DM1, it is rational to ask if the cytokines-related processes and pathways mediated both the defective myogenesis in DM1 and the rescue function of miR-322/-503.

### TNF Signaling Pathway Is Essential for DM1 Myogenesis

**Figure 5:**
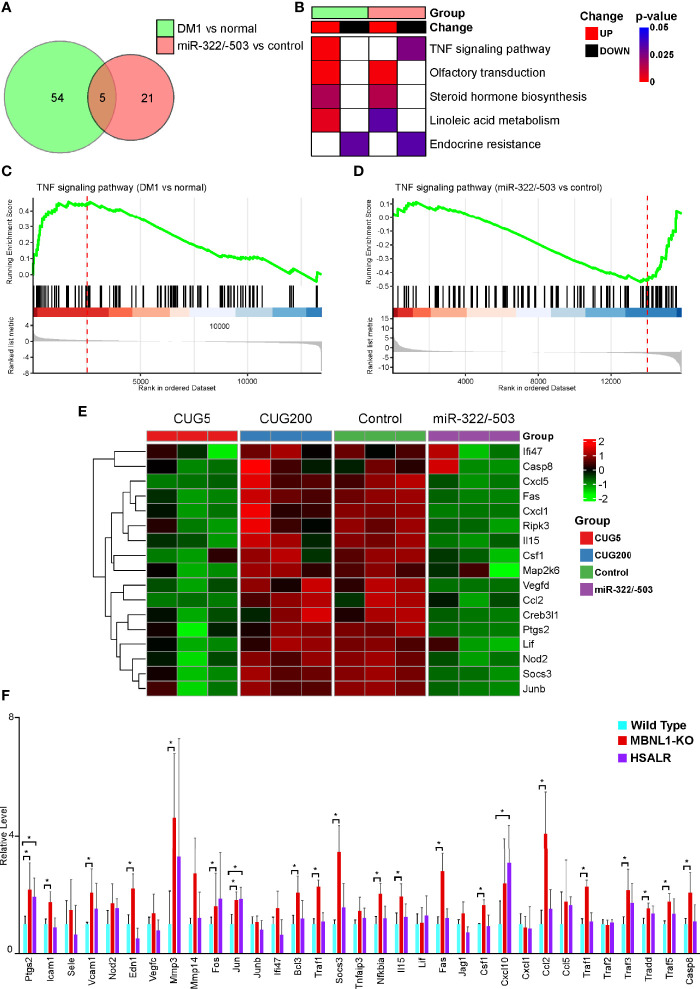
The TNF signal was tightly associated with DM1 myogenesis. GSEA was performed on the RNA-seq datasets of DM1 versus normal myoblasts and miR-322/-503 overexpression versus control DM1 myoblasts to find their significantly altered pathways, respectively. **(A)** Venn diagram of the pathways significantly altered in the two RNA-seq sets. **(B)** A heatmap showing the significance and change trends of the five signaling pathways shared by the two RNA-seq sets. **(C, D)**. The GSEA curves of the TNF signaling pathway in the two RNA-seq sets. **(E)** A heatmap showing the relative levels of TNF signaling-related genes in the two RNA-seq sets. **(F)** The relative levels of TNF signaling-related genes in the quadriceps muscles of wild-type, MBNL1 knockout, and HSALR mice. DM1 vs normal, the RNA-seq dataset of DM1 versus normal myoblasts; miR-322/-503 vs control, the RNA-seq dataset of miR-322/-503 overexpression versus control DM1 myoblasts; CUG5, the normal group; CUG200, the DM1 group; Control, control empty vector stably transfected DM1 myoblasts; miR-322/-503, miR-322/-503 overexpressing DM1 myoblasts; n≥3; *, p < 0.05.

To further explore which cytokine-related signaling pathway is involved in regulating DM1 myogenesis, we performed GSEA on the above two RNA-seq datasets. There were 54 significantly altered signaling pathways when comparing DM1 myoblasts to normal control, while 21 differential pathways with miR-322/-503 overexpression treatment on DM1 myoblasts (**Figure 5A**). Among these, there were five pathways changed in both datasets, which were TNF signaling pathway, Olfactory transduction, Steroid hormone biosynthesis, Linoleic acid metabolism, and Endocrine resistance. Considering that myogenesis was conversely regulated, it is logical to locate oppositely changed pathways as potential underlying mechanisms between these two experiment sets. Surprisingly, only TNF signaling pathway is hyperactivated in the DM1 model and inhibited by miR-322/-503 (**Figure 5B**). GSEA plots showed a clear upregulation of the TNF signaling pathway in the DM1 compared to the normal control, while a remarkable suppression with the miR-322/-503 treatment (**Figures 5C, D**). Furthermore, we analyzed the relative levels of TNF signaling-related genes in the differentiating normal myoblasts, DM1 myoblasts, miR-322/-503 overexpressing DM1 myoblasts, and its corresponding control myoblasts. These TNF signaling-related genes, such as Cxcl5, Fas, and Junb, were synchronously upregulated in the DM1 versus the normal group. With miR-322/-503 overexpression, however, these genes in DM1 myoblasts were significantly reduced back to normal levels (**Figure 5E**). To further verify these findings, we analyzed the expressions of TNF signaling-related genes in the RNA-seq data of quadricep muscles from the normal and DM1 (including MBNL1 knockout and HSALR mice) mouse models. As expected, both DM1 mouse models displayed upregulations of most TNF signaling-related genes (**Figure 5F**). These results encouraged us to investigate the potential function of the TNF signaling pathway in DM1 myogenesis.

### TNF Signaling Is Closely Associated With the Myogenesis Defects in DM1

**Figure 6:**
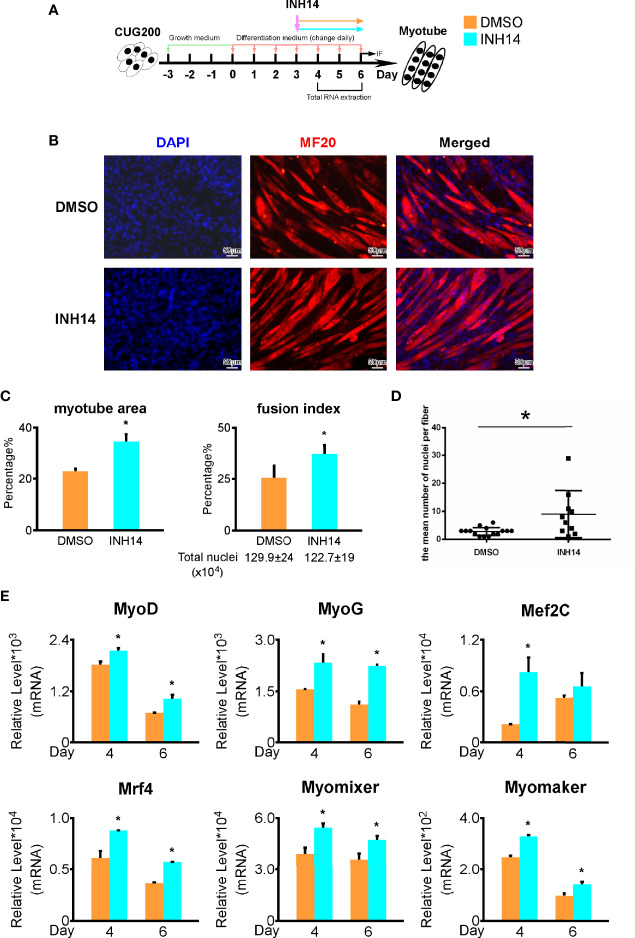
TNF inhibition rescued the DM1 myogenesis defects. **(A)** 100 nM INH14 (a TNF signaling inhibitor) was added to the DM1 myoblast differentiation to inhibit the TNF signaling from differentiation day 3 to day 6. DMSO was used as a control. **(B)** The INH14 treatment improved myotube formation in DM1 myoblasts. Immunofluorescence staining against MF20 was performed on differentiation day 6. **(C)** The myotube area and fusion index were significantly increased with the INH14 treatment. **(D)** The mean number of nuclei per fiber was significantly increased with the INH14 treatment. **(E)** The expression of muscle regulatory factors (MyoD, MyoG, Mef2C, and Mrf4) and myoblast fusion genes (Myomixer and Myomaker) was significantly upregulated with the INH14 treatment. DMSO, DMSO treatment control; INH14, INH14 treatment; n≥3; *, p < 0.05.

As TNF signaling is hyperactivated in DM1 myogenesis, we asked if inhibiting TNF signaling could rescue the DM1 myogenesis defects. We performed *in vitro* myoblast differentiation on DM1 myoblasts and inhibited TNF signaling pathway using 100 nM INH14 from differentiation day 3 to day 6 (**Figure 6A**). By immunostaining against MF20, we found that the INH14 group had more myotube formation (**Figure 6B**). The myotube area, fusion index, and mean number of nuclei per fiber in the INH14 group were all significantly upregulated (**Figures 6C, D**). By RT-qPCR, we found that myogenesis-related genes (MyoG, MyoD, Mef2C, Mrf4) and myoblast fusion markers (Myomixer, Myomaker) were all significantly upregulated (**Figure 6E**). We also investigated the effect of a higher INH14 concentration (15 μM) on DM1 myogenesis and found the myotube area, fusion index, and mean number of nuclei per fiber were all significantly upregulated in the INH14 group, either (**Figures S4A–C**). These results suggested that inhibiting TNF signaling pathway could improve myogenesis in DM1.

**Figure 7:**
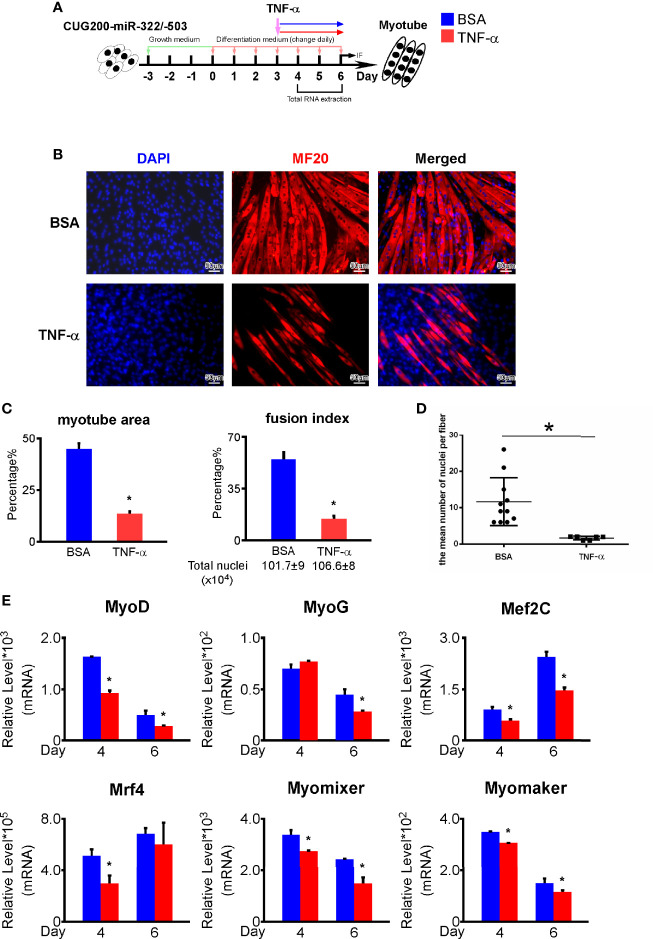
TNF-α treatment repressed the rescue function of miR-322/-503 on DM1 myogenesis. **(A)** 50 ng/ml TNF-α was added to the miR-322/-503 overexpressing DM1 myoblast differentiation from differentiation day 3 to day 6. 0.1% BSA was used as a control. **(B)** The TNF-α treatment impaired myotube formation in miR-322/-503 overexpressing DM1 myoblasts. Immunofluorescence staining against MF20 was performed on differentiation day 6. **(C)** The myotube area and fusion index were significantly decreased with the TNF-α treatment. **(D)** The mean number of nuclei per fiber was significantly decreased with the TNF-α treatment. **(E)** The expression of muscle regulatory factors (MyoD, MyoG, Mef2C, and Mrf4) and myoblast fusion genes (Myomixer and Myomaker) was significantly downregulated with the TNF-α treatment. BSA, BSA treatment control; TNF-α, TNF-α treatment; n≥3; *, p < 0.05.

As shown above, that TNF signaling was suppressed by miR-322/-503 in DM1 myoblasts, we asked if miR-322/-503 rescued DM1 myogenesis through inhibiting TNF signaling pathway. We boosted the TNF pathway by treating differentiating miR-322/-503 overexpressing DM1 myoblasts with TNF-α from differentiation day 3 to day 6 (**Figure 7A**). By immunostaining against MF20, we noticed a remarkable decrease of myotube formation and reduced myotube area, fusion index, and mean number of nuclei per fiber (**Figures 7B–D**). By RT-qPCR, we found that myogenesis-related genes (MyoG, MyoD, Mef2C, Mrf4) and myoblast fusion markers (Myomixer, Myomaker) were also significantly downregulated (**Figure 7E**). These results indicated that miR-322/-503 rescued DM1 myogenesis at least partially through inhibiting the TNF signaling pathway.

## Discussion

DM1 is an autosomal dominant inherited neuromuscular disease that is usually accompanied by defective myogenesis (30, 31). Our previous for the first time revealed the rescue function of miR-322/-503 on DM1 myoblast defects, including the defective myogenesis (13). However, the direct downstream pathway that mediates the rescue function of miR-322/-503 on DM1 myogenesis was still unclear. In this study, we found that the TNF signaling pathway, hyperactivated in DM1 myoblasts, was inhibited by miR-322/-503 and at least partially accounted for the rescue function of miR-322/-503 on DM1 myogenesis. Moreover, direct inhibiting the TNF pathway using chemical inhibitors also significantly improved DM1 myogenesis, which implied the TNF pathway as a druggable therapeutic target.

Based on the pathogenesis of DM1, most DM1 cell and animal models were produced by knockout MBNL1 or introducing ectopic expanded CTG repeats expression (32, 33). In this study, 200 CTG repeats were transfected into C2C12 cells to simulate DM1 defects, with 5 CTG repeats as control. As expected, compared with the control group, the expression of myogenic factors was significantly decreased in the DM1 group, with MyoD representing early-stage and MyoG and Mef2C representing middle and late stage (34, 35). Meanwhile, RNA-seq also found a significant decrease in muscle development-related processes in the DM1 group compared to the normal group. These results suggested that our DM1 myoblast model could reproduce the myogenesis defect. Our previous work revealed that miR-322/-503 can directly target CUG repeats to rescue myoblast defects (13). We first validated the function of miR-322/-503 on DM1 myogenesis through *in vitro* myoblast differentiation. Subsequently, by RNA-seq, we confirmed that miR-322/-503 could rescue muscle development in DM1.

Next, we investigated the underlying mechanisms that mediated both the defective myogenesis in DM1 and the rescue function of miR-322/-503. By analyzing RNA-seq data, we found five pathways that were both aberrantly altered in the DM1 group compared with the control group and significantly changed with miR-322/-503 treatment, which were TNF signaling pathway, Olfactory transduction, Steroid hormone biosynthesis, Linoleic acid metabolism, and Endocrine resistance. Among these, Endocrine resistance was the only signal inhibited in DM1. A previous study reported that endocrine resistance was significantly repressed with mTOR inhibitor treatment in breast cancer (36). As mTOR signaling was repressed in DM1 (37), it is logical to think that the inhibition of endocrine resistance might be caused by the change of mTOR signaling. In addition, the decreased endocrine resistance might imply a therapeutic role of insulin in DM1, since insulin is a classical mTOR signaling activator (38). The enhancement of olfactory transduction might affect autophagy. Studies have found that olfactory receptor 544 promoted the expression of LC3 in skeletal muscles (39), which was consistent with the enhancement of autophagy in DM1 (40). As a steroid hormone, vitamin D played a direct regulatory role in skeletal muscle development, participating in myogenesis, cell proliferation, and differentiation (41). Previous reports have revealed that an active form of Vitamin D3 inhibited the myoblast differentiation of C2C12 by activating the Erk1/2 signaling (42). The up-regulation of linoleic acid metabolism was correlated with the TNF signaling. As previously reported, conjugated linoleic acid reduced the cell death of C2C12 cells under TNF-α treatment (43), indicating that DM1 produced self-rescue signals in response to the inflammatory environment. Apart from these, the TNF signaling was the only pathway significantly hyperactivated in the DM1 myoblast and inhibited by miR-322/-503. The hyperactivation of TNF signaling in DM1 was validated in the DM1 mice model as well.

Previous studies on TNF signaling mainly focused on inflammatory responses (44), suggesting an association between inflammatory activation and DM1 disease. Although Inflammatory activation has been reported to activate myogenic cell proliferation and differentiation during the initial phase of muscle repair (45), inhibition of inflammatory CCR2 signaling significantly promoted muscle regeneration during a post-injury repair (46). Similarly, we here found that inhibition of the inflammatory cytokine TNF signaling rescued the DM1 myogenesis defect. In addition, TNF-α has been reported to induce apoptosis and autophagy of C2C12 cells by activating NF-κB signaling (20, 47), while knockdown TNF reduces insulin resistance in C2C12 cells (48). Consistently, in this study, TNF-α treatment significantly impaired myoblast differentiation in the miR-322/-503 rescue model. This may suggest that inflammatory response might be responsible for DM1 myoblast differentiation defects, and that inhibition of its activity could rescue muscle development.

Myogenesis from myoblasts includes three sequential stages: cell cycle exit, cell alignment, and fusion. Cell cycle exit is the prerequisite that ensures the production of myocytes from myoblasts. Through Ki67 immunostaining, we found that DM1 myoblasts had a higher proliferation rate than the normal control. This agreed with a previous report that overexpressing Celf1, which is aberrantly upregulated in DM1, promoted myoblast proliferation (49). Moreover, we revealed that DM1 myoblasts retained a hyperactive TNF signaling, which might be at least partially responsible for the defective myogenesis of DM1. A previous study showed that activating TNF signaling using TNF-α could increase NF-κB activation and promote the proliferation of primary mouse muscle precursor cells and C2C12 cells (50). These results together might imply that DM1 myoblasts had an impaired cell cycle exit ability that might be at least partially caused by the hyperactive TNF signaling. On the contrary, miR-322/-503 might promote the cell cycle exit stage of the myogenesis process by inhibiting the TNF signaling. This was in line with a report that miR-322/424 and miR-503 initiate normal muscle differentiation by promoting cell cycle quiescence (51). To sum up, miR-322/-503 inhibiting the TNF signaling promoted the cell cycle exit of DM1 myoblasts, which favored the myogenesis in DM1.

Our study demonstrated that the TNF signaling might be responsible for the defective myogenesis in DM1 and at least partially mediate the rescue function of miR-322/-503 on DM1 myogenesis. Blocking TNF signaling using chemical inhibitors significantly improved the myogenesis in DM1, implying a potential therapeutic approach against DM1 muscle wasting.

## Data Availability Statement

The datasets presented in this study can be found in online repositories. The names of the repository/repositories and accession number(s) can be found below: GEO [accession: GSE174119, GSE189897].

## Author Contributions

Conceptualization, XS, GZ, and ML. Methodology, XS, GZ, ML, and FX. Investigation, ML and FX. Formal analysis, ML, ZL, CW, and YZ. Writing – original draft, ML. Writing – review and editing, XS, GZ, and ML. Supervision, XS and GZ. All authors contributed to the article and approved the submitted version.

## Funding

This work was supported by the National Natural Science Foundation of China (No. 31701289), Anhui Provincial Natural Science Foundation (No. 1808085QH234), Anhui Provincial Funding Scheme to Outstanding Innovative Programs by Returned Scholars (No. 2019LCX003), Anhui Provincial Key Laboratory of Molecular Enzymology and Mechanism of Major Diseases (No. fzmx202001), Educational Commission of Anhui Province of China (No. KJ2017A319, KJ2019A0498, KJ2020A0058, KJ2020A0087), Key Projects for Young and Middle-Aged People from Wannan Medical College (No. WK2021ZF08), and the Foundation for High-level Talents in Higher Education of Anhui Province of China and Funds from the Anhui Normal University (No. 2017XJJ38, start-up funds to XS).

## Conflict of Interest

The authors declare that the research was conducted in the absence of any commercial or financial relationships that could be construed as a potential conflict of interest.

## Publisher’s Note

All claims expressed in this article are solely those of the authors and do not necessarily represent those of their affiliated organizations, or those of the publisher, the editors and the reviewers. Any product that may be evaluated in this article, or claim that may be made by its manufacturer, is not guaranteed or endorsed by the publisher.

## Supplementary Material

Supplementary Figure 1 Immunostaining against Ki67 on the normal and DM1 myoblasts. CUG5, the normal group; CUG200, the DM1 group; n≥3.

Supplementary Figure 2 Annexin V/PI apoptosis assays on the normal and DM1 myoblasts. CUG5, the normal group; CUG200, the DM1 group; n≥3.

Supplementary Figure 3 The levels of ECM related and cytokine factors along the differentiation of the normal and DM1 myoblasts. RT-qPCR was performed on ECM-related factors (Col1a1, Fmod, Postn) and cytokine factors (Cxcl5, Ccl2, IL1β). These genes were all significantly upregulated during DM1 myogenesis. CUG5, the normal group; CUG200, the DM1 group; n≥3; *, p < 0.05.

Supplementary Figure 4 15 μM INH14 treatment also rescued the DM1 myogenesis defects. **(A)** 15 μM INH14 treatment improved myotube formation in DM1 myoblasts. Immunofluorescence staining against MF20 was performed on differentiation day 6. (B) The myotube area and fusion index were significantly increased with 15 μM INH14 treatment. (C) The mean number of nuclei per fiber was significantly increased with 15 μM INH14 treatment. DMSO, DMSO treatment control; 15 μM INH14, 15 μM INH14 treatment; n≥3; *, p < 0.05.

## References

[1] Filippova GN Thienes CP Penn BH Cho DH Hu YJ Moore JM. Ctcf-Binding Sites Flank Ctg/Cag Repeats and Form a Methylation-Sensitive Insulator at the Dm1 Locus. Nat Genet (2001) 28(4):335–43. doi: 10.1038/ng570 11479593

[2] Yin Q Wang H Li N Ding Y Xie Z Jin L. Dosage Effect of Multiple Genes Accounts for Multisystem Disorder of Myotonic Dystrophy Type 1. Cell Res (2020) 30(2):133–45. doi: 10.1038/s41422-019-0264-2 PMC7015062 31853004

[3] Lopez Castel A Cleary JD Pearson CE. Repeat Instability as the Basis for Human Diseases and as a Potential Target for Therapy. Nat Rev Mol Cell Biol (2010) 11(3):165–70. doi: 10.1038/nrm2854 20177394

[4] Dean NL Tan SL Ao A. Instability in the Transmission of the Myotonic Dystrophy Ctg Repeat in Human Oocytes and Preimplantation Embryos. Fertil Steril (2006) 86(1):98–105. doi: 10.1016/j.fertnstert.2005.12.025 16716318

[5] Chal J Pourquie O. Making Muscle: Skeletal Myogenesis in Vivo and in Vitro. Development (2017) 144(12):2104–22. doi: 10.1242/dev.151035 28634270

[6] Zammit PS Golding JP Nagata Y Hudon V Partridge TA Beauchamp JR. Muscle Satellite Cells Adopt Divergent Fates: A Mechanism for Self-Renewal? J Cell Biol (2004) 166(3):347–57. doi: 10.1083/jcb.200312007 PMC2172269 15277541

[7] Bentzinger CF Wang YX Rudnicki MA. Building Muscle: Molecular Regulation of Myogenesis. Cold Spring Harb Perspect Biol (2012) 4(2):a008342. doi: 10.1101/cshperspect.a008342 22300977 PMC3281568

[8] Cheng X Du J Shen L Tan Z Jiang D Jiang A. Mir-204-5p Regulates C2c12 Myoblast Differentiation by Targeting Mef2c and Errgamma. BioMed Pharmacother (2018) 101:528–35. doi: 10.1016/j.biopha.2018.02.096 29505923

[9] Ma Z Sun X Xu D Xiong Y Zuo B. Microrna, Mir-374b, Directly Targets Myf6 and Negatively Regulates C2c12 Myoblasts Differentiation. Biochem Biophys Res Commun (2015) 467(4):670–5. doi: 10.1016/j.bbrc.2015.10.086 26498529

[10] Petrany MJ Millay DP. Cell Fusion: Merging Membranes and Making Muscle. Trends Cell Biol (2019) 29(12):964–73. doi: 10.1016/j.tcb.2019.09.002 PMC7849503 31648852

[11] Huang B Jiao Y Zhu Y Ning Z Ye Z Li QX. Mdfi Promotes C2c12 Cell Differentiation and Positively Modulates Fast-To-Slow-Twitch Muscle Fiber Transformation. Front Cell Dev Biol (2021) 9:605875. doi: 10.3389/fcell.2021.605875 33553177 PMC7862576

[12] Koscianska E Witkos TM Kozlowska E Wojciechowska M Krzyzosiak WJ. Cooperation Meets Competition in Microrna-Mediated Dmpk Transcript Regulation. Nucleic Acids Res (2015) 43(19):9500–18. doi: 10.1093/nar/gkv849 PMC4627076 26304544

[13] Shen X Xu F Li M Wu S Zhang J Wang A. Mir-322/-503 Rescues Myoblast Defects in Myotonic Dystrophy Type 1 Cell Model by Targeting Cug Repeats. Cell Death Dis (2020) 11(10):891. doi: 10.1038/s41419-020-03112-6 33093470 PMC7582138

[14] Kalliolias GD Ivashkiv LB. Tnf Biology, Pathogenic Mechanisms and Emerging Therapeutic Strategies. Nat Rev Rheumatol (2016) 12(1):49–62. doi: 10.1038/nrrheum.2015.169 26656660 PMC4809675

[15] Hayden MS Ghosh S. Regulation of Nf-Kappab by Tnf Family Cytokines. Semin Immunol (2014) 26(3):253–66. doi: 10.1016/j.smim.2014.05.004 PMC4156877 24958609

[16] Wang L Du F Wang X. Tnf-Alpha Induces Two Distinct Caspase-8 Activation Pathways. Cell (2008) 133(4):693–703. doi: 10.1016/j.cell.2008.03.036 18485876

[17] Li M Guo Q Cai H Wang H Ma Z Zhang X. Mir-218 Regulates Diabetic Nephropathy *Via* Targeting Ikk-Beta and Modulating Nk-Kappab-Mediated Inflammation. J Cell Physiol (2020) 235(4):3362–71. doi: 10.1002/jcp.29224 PMC13482667 31549412

[18] Nan Y Guo L Zhen Y Wang L Ren B Chen X. Mirna-451 Regulates the Nf-Kappab Signaling Pathway by Targeting Ikkbeta to Inhibit Glioma Cell Growth. Cell Cycle (2021) 20(19):1967–77. doi: 10.1080/15384101.2021.1969496 PMC8565813 34463194

[19] Gu H Yu J Dong D Zhou Q Wang JY Yang P. The Mir-322-Traf3 Circuit Mediates the Pro-Apoptotic Effect of High Glucose on Neural Stem Cells. Toxicol Sci (2015) 144(1):186–96. doi: 10.1093/toxsci/kfu271 PMC4349142 25516495

[20] Zhao Q Yang ST Wang JJ Zhou J Xing SS Shen CC. Tnf Alpha Inhibits Myogenic Differentiation of C2c12 Cells Through Nf-Kappab Activation and Impairment of Igf-1 Signaling Pathway. Biochem Biophys Res Commun (2015) 458(4):790–5. doi: 10.1016/j.bbrc.2015.02.026 25686491

[21] Li J Yi X Yao Z Chakkalakal JV Xing L Boyce BF. Tnf Receptor-Associated Factor 6 Mediates Tnfalpha-Induced Skeletal Muscle Atrophy in Mice During Aging. J Bone Miner Res (2020) 35(8):1535–48. doi: 10.1002/jbmr.4021 PMC7429284 32267572

[22] Meyer SU Sass S Mueller NS Krebs S Bauersachs S Kaiser S. Integrative Analysis of Microrna and Mrna Data Reveals an Orchestrated Function of Micrornas in Skeletal Myocyte Differentiation in Response to Tnf-Alpha or Igf1. PloS One (2015) 10(8):e0135284. doi: 10.1371/journal.pone.0135284 26270642 PMC4536022

[23] Amack JD Mahadevan MS. The Myotonic Dystrophy Expanded Cug Repeat Tract Is Necessary But Not Sufficient to Disrupt C2c12 Myoblast Differentiation. Hum Mol Genet (2001) 10(18):1879–87. doi: 10.1093/hmg/10.18.1879 11555624

[24] Rueden CT Schindelin J Hiner MC DeZonia BE Walter AE Arena ET. Imagej2: Imagej for the Next Generation of Scientific Image Data. BMC Bioinf (2017) 18(1):529. doi: 10.1186/s12859-017-1934-z PMC5708080 29187165

[25] Kim D Langmead B Salzberg SL. Hisat: A Fast Spliced Aligner With Low Memory Requirements. Nat Methods (2015) 12(4):357–60. doi: 10.1038/nmeth.3317 PMC4655817 25751142

[26] Pertea M Pertea GM Antonescu CM Chang TC Mendell JT Salzberg SL. Stringtie Enables Improved Reconstruction of a Transcriptome From Rna-Seq Reads. Nat Biotechnol (2015) 33(3):290–5. doi: 10.1038/nbt.3122 PMC4643835 25690850

[27] Love MI Huber W Anders S. Moderated Estimation of Fold Change and Dispersion for Rna-Seq Data With Deseq2. Genome Biol (2014) 15(12):550. doi: 10.1186/s13059-014-0550-8 25516281 PMC4302049

[28] Yu G Wang LG Han Y He QY. Clusterprofiler: An R Package for Comparing Biological Themes Among Gene Clusters. OMICS (2012) 16(5):284–7. doi: 10.1089/omi.2011.0118 PMC3339379 22455463

[29] Du H Cline MS Osborne RJ Tuttle DL Clark TA Donohue JP. Aberrant Alternative Splicing and Extracellular Matrix Gene Expression in Mouse Models of Myotonic Dystrophy. Nat Struct Mol Biol (2010) 17(2):187–93. doi: 10.1038/nsmb.1720 PMC2852634 20098426

[30] Huguet A Medja F Nicole A Vignaud A Guiraud-Dogan C Ferry A. Molecular, Physiological, and Motor Performance Defects in Dmsxl Mice Carrying >1,000 Ctg Repeats From the Human Dm1 Locus. PloS Genet (2012) 8(11):e1003043. doi: 10.1371/journal.pgen.1003043 23209425 PMC3510028

[31] Udd B Krahe R. The Myotonic Dystrophies: Molecular, Clinical, and Therapeutic Challenges. Lancet Neurol (2012) 11(10):891–905. doi: 10.1016/S1474-4422(12)70204-1 22995693

[32] Kanadia RN Johnstone KA Mankodi A Lungu C Thornton CA Esson D. A Muscleblind Knockout Model for Myotonic Dystrophy. Science (2003) 302(5652):1978–80. doi: 10.1126/science.1088583 14671308

[33] Lueck JD Mankodi A Swanson MS Thornton CA Dirksen RT. Muscle Chloride Channel Dysfunction in Two Mouse Models of Myotonic Dystrophy. J Gen Physiol (2007) 129(1):79–94. doi: 10.1085/jgp.200609635 17158949 PMC2151606

[34] Zammit PS. Function of the Myogenic Regulatory Factors Myf5, Myod, Myogenin and Mrf4 in Skeletal Muscle, Satellite Cells and Regenerative Myogenesis. Semin Cell Dev Biol (2017) 72:19–32. doi: 10.1016/j.semcdb.2017.11.011 29127046

[35] Liu N Nelson BR Bezprozvannaya S Shelton JM Richardson JA Bassel-Duby R. Requirement of Mef2a, C, and D for Skeletal Muscle Regeneration. Proc Natl Acad Sci USA (2014) 111(11):4109–14. doi: 10.1073/pnas.1401732111 PMC3964114 24591619

[36] Beeram M Tan QT Tekmal RR Russell D Middleton A DeGraffenried LA. Akt-Induced Endocrine Therapy Resistance Is Reversed by Inhibition of Mtor Signaling. Ann Oncol (2007) 18(8):1323–8. doi: 10.1093/annonc/mdm170 17693645

[37] Denis JA Gauthier M Rachdi L Aubert S Giraud-Triboult K Poydenot P. Mtor-Dependent Proliferation Defect in Human Es-Derived Neural Stem Cells Affected by Myotonic Dystrophy Type 1. J Cell Sci (2013) 126(Pt 8):1763–72. doi: 10.1242/jcs.116285 23444380

[38] Renna LV Bose F Brigonzi E Fossati B Meola G Cardani R. Aberrant Insulin Receptor Expression Is Associated With Insulin Resistance and Skeletal Muscle Atrophy in Myotonic Dystrophies. PloS One (2019) 14(3):e0214254. doi: 10.1371/journal.pone.0214254 30901379 PMC6430513

[39] Thach TT Wu C Hwang KY Lee SJ. Azelaic Acid Induces Mitochondrial Biogenesis in Skeletal Muscle by Activation of Olfactory Receptor 544. Front Physiol (2020) 11:329. doi: 10.3389/fphys.2020.00329 32411005 PMC7199515

[40] Bargiela A Cerro-Herreros E Fernandez-Costa JM Vilchez JJ Llamusi B Artero R. Increased Autophagy and Apoptosis Contribute to Muscle Atrophy in a Myotonic Dystrophy Type 1 Drosophila Model. Dis Model Mech (2015) 8(7):679–90. doi: 10.1242/dmm.018127 PMC4486854 26092529

[41] Montenegro KR Cruzat V Carlessi R Newsholme P. Mechanisms of Vitamin D Action in Skeletal Muscle. Nutr Res Rev (2019) 32(2):192–204. doi: 10.1017/S0954422419000064 31203824

[42] Wang Z Jiang A Mei J Zhang X. 1alpha,25(Oh)2-Vitamin D3 Inhibits C2c12 Cell Differentiation by Activating C-Src and Erk1/2. Clin Lab (2018) 64(5):687–98. doi: 10.7754/Clin.Lab.2017.171016 29739041

[43] Mohammadi I Mahdavi AH Rabiee F Nasr Esfahani MH Ghaedi K. Positive Effects of Conjugated Linoleic Acid (Cla) on the Pgc1-Alpha Expression Under the Inflammatory Conditions Induced by Tnf-Alpha in the C2c12 Cell Line. Gene (2020) 735:144394. doi: 10.1016/j.gene.2020.144394 31987906

[44] Bradley JR. Tnf-Mediated Inflammatory Disease. J Pathol (2008) 214(2):149–60. doi: 10.1002/path.2287 18161752

[45] Charge SB Rudnicki MA. Cellular and Molecular Regulation of Muscle Regeneration. Physiol Rev (2004) 84(1):209–38. doi: 10.1152/physrev.00019.2003 14715915

[46] Blanc RS Kallenbach JG Bachman JF Mitchell A Paris ND Chakkalakal JV. Inhibition of Inflammatory Ccr2 Signaling Promotes Aged Muscle Regeneration and Strength Recovery After Injury. Nat Commun (2020) 11(1):4167. doi: 10.1038/s41467-020-17620-8 32820177 PMC7441393

[47] Guttridge DC Mayo MW Madrid LV Wang CY Baldwin AS Jr. Nf-Kappab-Induced Loss of Myod Messenger Rna: Possible Role in Muscle Decay and Cachexia. Science (2000) 289(5488):2363–6. doi: 10.1126/science.289.5488.2363 11009425

[48] Haghani K Pashaei S Vakili S Taheripak G Bakhtiyari S. Tnf-Alpha Knockdown Alleviates Palmitate-Induced Insulin Resistance in C2c12 Skeletal Muscle Cells. Biochem Biophys Res Commun (2015) 460(4):977–82. doi: 10.1016/j.bbrc.2015.03.137 25839650

[49] Peng X Shen X Chen X Liang R Azares AR Liu Y. Celf1 Regulates Cell Cycle and Is Partially Responsible for Defective Myoblast Differentiation in Myotonic Dystrophy Rna Toxicity. Biochim Biophys Acta (2015) 1852(7):1490–7. doi: 10.1016/j.bbadis.2015.04.010 25887157

[50] Otis JS Niccoli S Hawdon N Sarvas JL Frye MA Chicco AJ. Pro-Inflammatory Mediation of Myoblast Proliferation. PloS One (2014) 9(3):e92363. doi: 10.1371/journal.pone.0092363 24647690 PMC3960233

[51] Sarkar S Dey BK Dutta A. Mir-322/424 and -503 Are Induced During Muscle Differentiation and Promote Cell Cycle Quiescence and Differentiation by Down-Regulation of Cdc25a. Mol Biol Cell (2010) 21(13):2138–49. doi: 10.1091/mbc.E10-01-0062 PMC2893979 20462953

