# Supplementary material for: TNF Signaling Acts Downstream of MiR-322/-503 in Regulating DM1 Myogenesis
Source: Front Endocrinol (Lausanne). 2022 Apr 7;13:843202. doi: 10.3389/fendo.2022.843202 (PMC9021394; doi:10.3389/fendo.2022.843202)
Supplement: Supplementary Figure 1 — Immunostaining against Ki67 on the normal and DM1 myoblasts. CUG5, the normal group; CUG200, the DM1 group; n≥3. [file Presentation_1.pdf]

SUPPLEMENTARY FIGURES AND LEGENDS

Figure S1. Immunostaining against Ki67 on the normal and DM1 myoblasts. CUG5, the normal group; CUG200, the DM1 group; n≥3.

Figure S2. Annexin V /PI apoptosis assays on the normal and DM1 myoblasts. CUG5, the normal group; CUG200, the DM1 group; n≥3.

Figure S3. The levels of ECM related and cytokine factors along the differentiation of the normal and DM1 myoblasts. RT-qPCR was performed on ECM-related factors (Col1a1, Fmod, Postn) and cytokine factors (Cxcl5, Ccl2, IL1β). These genes were all significantly upregulated during DM1 myogenesis. CUG5, the normal group; CUG200, the DM1 group; n≥3; *, $p < 0.05$.

Figure S4. 15 μM INH14 treatment also rescued the DM1 myogenesis defects. (A) 15 μM INH14 treatment improved myotube formation in DM1 myoblasts. Immunofluorescence staining against MF20 was performed on differentiation day 6. (**B**) The myotube area and fusion index were significantly increased with 15 μM INH14 treatment. (**C**) The mean number of nuclei per fiber was significantly increased with 15 μM INH14 treatment. DMSO, DMSO treatment control; 15 μM INH14, 15 μM INH14 treatment; n≥3; *, $p < 0.05$.

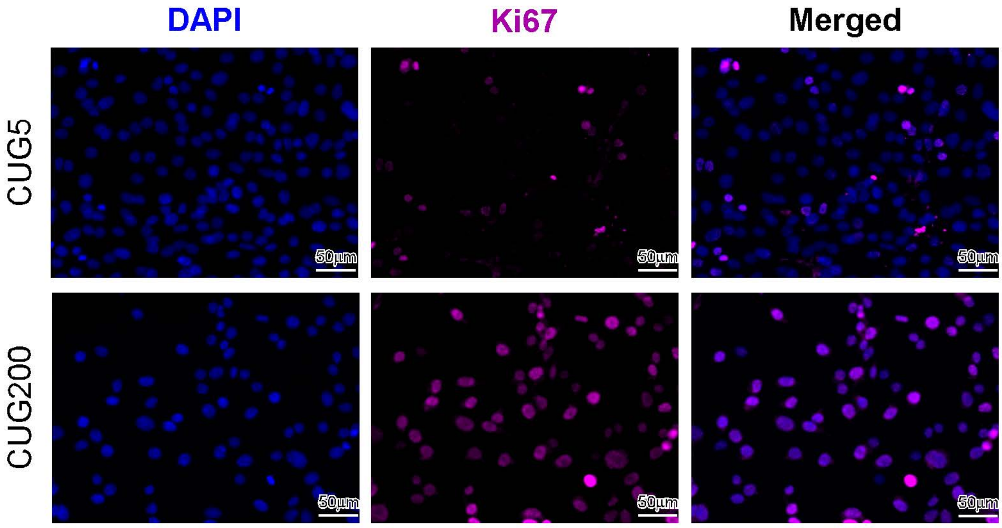

Figure S1

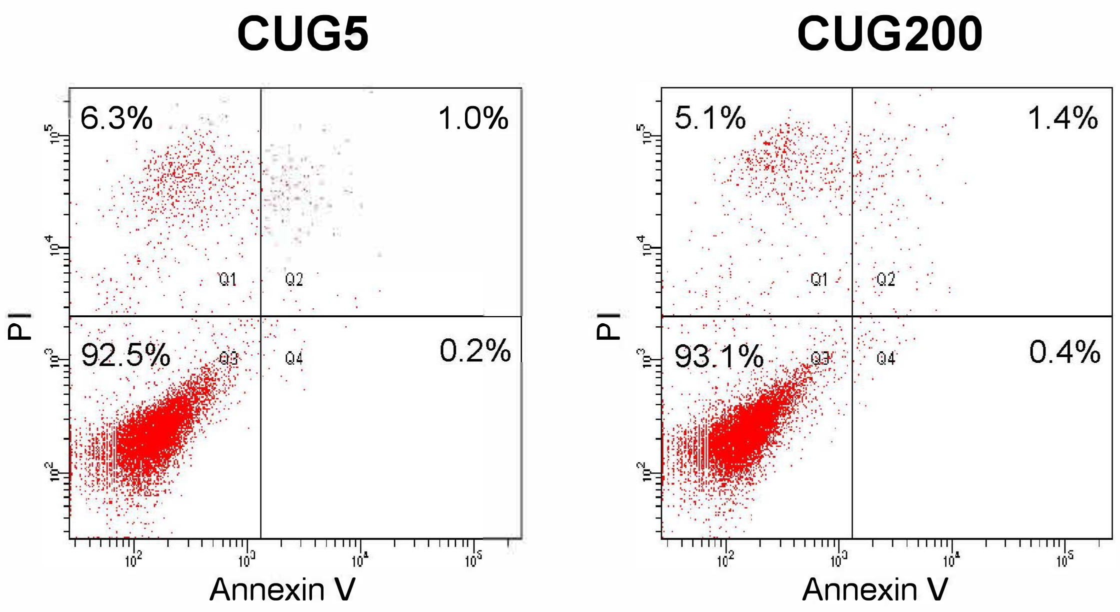

Figure S2

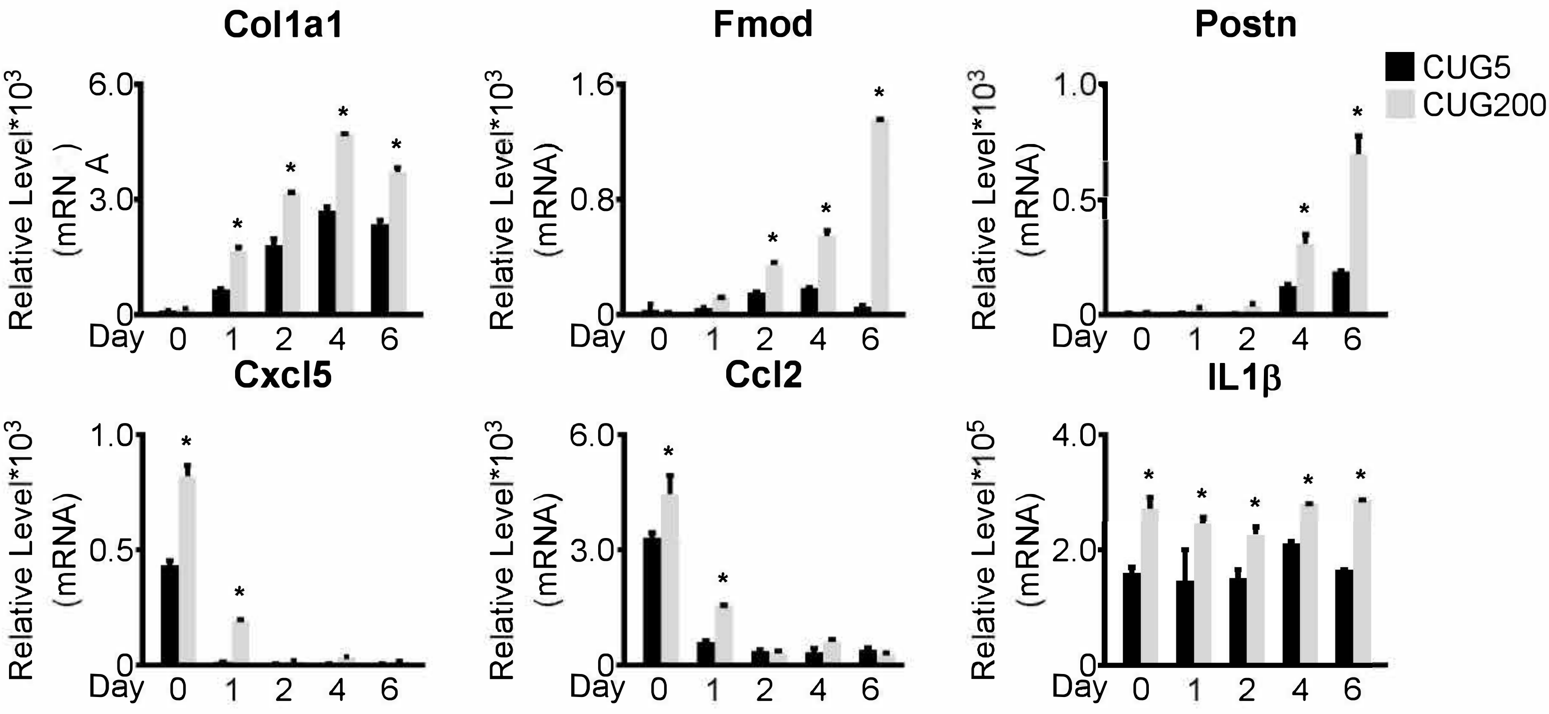
Col1a1
Fmod
Postn
CUG5
CUG200
Cxcl5
Ccl2
IL1β
Relative Level*10^3 (mRNA)
Relative Level*10^5 (mRNA)
Day
0
1
2
4
6
*

Figure S3

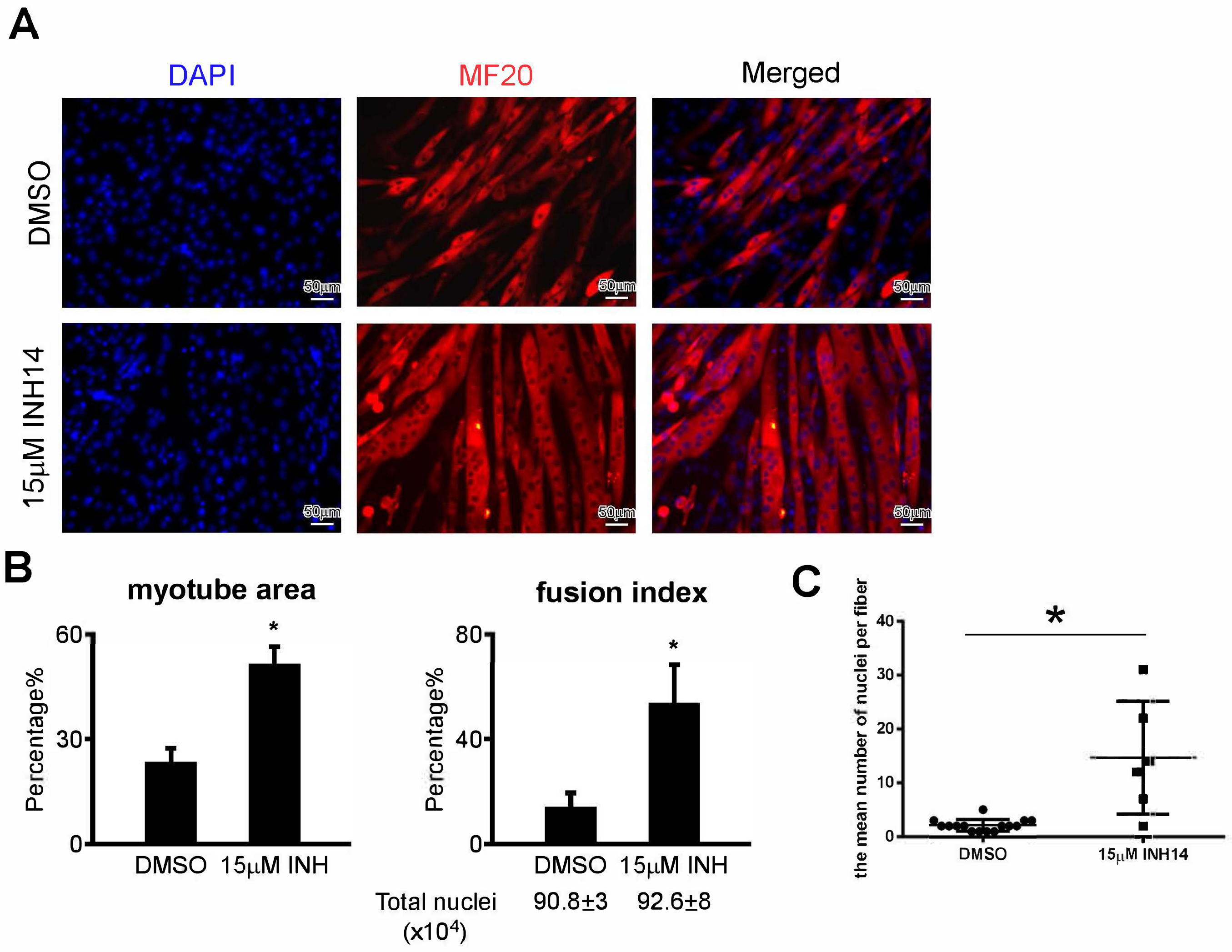
A
DAPI
MF20
Merged
DMSO
15µM INH14
50µm
B
myotube area
Percentage%
60
30
0
*
DMSO
15µM INH
fusion index
Percentage%
80
40
0
*
DMSO
15µM INH
Total nuclei
(x10⁴)
90.8±3
92.6±8
C
the mean number of nuclei per fiber
40
30
20
10
0
*
DMSO
15µM INH14

Figure S4
