# Supplementary material for: TNF Signaling Acts Downstream of MiR-322/-503 in Regulating DM1 Myogenesis
Source: Front Endocrinol (Lausanne). 2022 Apr 7;13:843202. doi: 10.3389/fendo.2022.843202 (PMC9021394; doi:10.3389/fendo.2022.843202)
Supplement: Supplementary file 2 [file Table_1.docx]

***Table S1. List of primers used for RT-qPCR***

| **Primers** | **Sequences(5’-3’)** |
| --- | --- |
| MyoD-F | ATGGCATGATGGATTACAGCGG |
| MyoD-R | CTATGCTGGACAGGCAGTCG |
| MyoG-F | ACTCCCTTACGTCCATCGTG |
| MyoG-R | CAGGACAGCCCCACTTAAAA |
| Mef2C-F | CGGTGTCGTCAGTTGTATGG |
| Mef2C-R | TGCAGTAGATATGCGGCTTG |
| Mrf4-F | ACTGCTAAGGAAGGAGGAGCA |
| Mrf4-R | CTGGCATCTGTGAGCCCTAA |
| Myomixer-F | CTGAGCAGTTCTGACTGGTG |
| Myomixer-R | CACCATCGGGAGCAATGGAA |
| Myomaker-F | ATCGCTACCAAGAGGCGTT |
| Myomaker-R | CACAGCACAGACAAACCAGG |
| Gapdh-F | CAAGCTCATTTCCTGGTATGACAA |
| Gapdh-R | GGGATAGGGCCTCTCTTGCT |
| Col1a1-F | CCAGGTCCCAAGGGTAACAG |
| Col1a1-R | ACTCCAGTAGCACCGGGTT |
| Fmod-F | GAACAGTTCAACCCAAGAGACA |
| Fmod-R | GGGGTCGTAGTAGGTGGACT |
| Postn-F | CCTGCCCTTATATGCTCTGCT |
| Postn-R | AAACATGGTCAATAGGCATCACT |
| Cxcl5-F | TGCCCTACGGTGGAAGTCAT |
| Cxcl5-R | AGCTTTCTTTTTGTCACTGCCC |
| Ccl2-F | CACTCACCTGCTGCTACTCA |
| Ccl2-R | GCTTGGTGACAAAAACTACAGC |
| IL1b-F | GTGTCTTTCCCGTGGACCTT |
| IL1b-R | AATGGGAACGTCACACACCA |
